# Association between Mid-Term Functionality and Clinical Severity in Patients Hospitalized for Pulmonary Embolism

**DOI:** 10.3390/healthcare12131323

**Published:** 2024-07-02

**Authors:** Ana Belén Gámiz-Molina, Geraldine Valenza-Peña, Julia Raya-Benítez, Alejandro Heredia-Ciuró, María Granados-Santiago, Laura López-López, Marie Carmen Valenza

**Affiliations:** 1 Pulmonology Service, San Cecilio University Hospital, 18016 Granada, Spain; abelen92@correo.ugr.es; 2 Department of Physiotherapy, Faculty of Health Sciences, University of Granada, 18071 Granada, Spain; geraldinevalenza@ugr.es (G.V.-P.); ahc@ugr.es (A.H.-C.); cvalenza@ugr.es (M.C.V.); 3 Nursing Department, Faculty of Health Sciences, University of Granada, 18071 Granada, Spain; juliarb@ugr.es (J.R.-B.); mariagranados@ugr.es (M.G.-S.)

**Keywords:** pulmonary embolism, clinical severity, quality of life, hospitalization, functionality

## Abstract

The aim of this study is to evaluate the relationship between clinical severity and functionality, occupational performance, and health-related quality of life in patients hospitalized with pulmonary embolism. Pulmonary embolism patients were grouped by clinical severity using the Pulmonary Embolism Severity Index. Those scoring ≥160 were in the high-severity group (HSG); those scoring < 160 in the low–moderate group (LMSG). The main variables were functionality assessed by the World Health Organization Disability Assessment Schedule (WHODAS), self-perception of occupational performance assessed by the Canadian Occupational Performance Measure (COPM), pain and fatigue assessed by a Visual Analogue Scale (VAS), and health-related quality of life assessed by the EuroQol-5Dimensions (EQ-5D). Patients were evaluated at hospital admission and at 1-month and 3-month follow-up. At admission, there were significant differences between groups in the WHODAS and health-related quality of life in favor of the LMSG. At 1-month and at 3-month follow-up, there were significant differences between the LMSG and HSG in WHODAS, COMP, NRS pain, fatigue and EQ-5D scores in favor of the LMSG. An association exists between clinical severity and mid-term functionality, self-perception of occupational performance, pain, fatigue, and health-related quality of life in PE patients.

## 1. Introduction

Pulmonary embolism, the most severe manifestation of venous thromboembolism, leads to the hospitalization or death of over 225,000 and 300,000 individuals annually in America and Europe, respectively [1]. These numbers have witnessed an increase over the past decade. The incidence of pulmonary embolism in the population varies from 21 to 69 cases per 100,000 per year, with an individual’s lifetime risk estimated at approximately 5% [2]. It ranks as the third leading cause of cardiovascular mortality, contributing to 5–10% of all deaths in North American hospitals [3].

Thanks to advancements in anticoagulation treatment, a substantial majority of pulmonary embolism patients now survive the acute event, with hemodynamically stable cases increasingly managed as outpatients [4]. Pulmonary embolism (PE) is a critical and potentially life-threatening condition with acute consequences. However, its long-term impact on the overall life and functionality of affected individuals has garnered significant attention in recent years [5].

The transition of patients with pulmonary embolism from the emergency department to hospitalization remains challenging due to the complexity of their clinical profiles. In line with this, different scores have been developed to aid medical decisions. More specifically, the Pulmonary Embolism Severity Index (PESI) serves as a specialized clinical decision tool intended to assist in this determination [6,7]. Its validation extends to outcomes such as 30-day mortality or severe complications, covering conditions like cardiogenic shock and cardio-respiratory arrest [8], as well as 90-day mortality [9]. While the PESI was developed as a decision-making aid, in recent years, numerous articles have utilized it as a prognostic tool for the acute management of PE. This is due to a growing recognition that the consequences of this thromboembolic event extend beyond the immediate clinical setting [10].

However, emerging evidence suggests that even after successful treatment, patients may experience a spectrum of persistent clinical sequelae [11]. These include but are not limited to chronic dyspnea, exercise intolerance, and lingering cardiovascular complications, known as the chronic sequelae of post-thrombotic syndrome [12].

Studies show that the prevalence and severity of these sequelae vary considerably and involve variable chronic manifestations that impact patients’ functional status and quality of life. Therefore, they are a crucial aspect that requires meticulous examination.

The impact of PE severity during hospitalization can extend to mid-term physical, emotional, and social well-being, posing persistent challenges to daily activities and interpersonal relationships. Understanding it can help elucidate potential therapeutic targets for PE survivors post-hospitalization.

By delving into the intricate interplay between the acute and post-hospitalization phases of PE, this study aims to shed light on the relationship between severity profile and mid-term post-PE outcomes, thereby elucidating its implications for patient clinical profiles.

## 2. Methods

### 2.1. Study Design

This was a prospective cohort study conducted from October 2022 to October 2023, involving patients admitted for pulmonary embolism (PE) under the care of the pulmonology department at San Cecilio Clinical University Hospital in Granada, Spain.

This study received approval from the Biomedical Research Ethical Committee of Granada (ID: 1770-N-23) and adhered to the ethical guidelines of the Declaration of Helsinki [13], last reviewed in 2013. The STROBE guidelines were followed during this research study [14].

### 2.2. Participants

Patients with PE were recruited during their hospitalization under the care of the pulmonology service. All subjects who voluntarily agreed to participate signed a written informed consent form after receiving detailed information about the study protocol.

The inclusion criteria for PE patients were as follows:

(a) stable patients with pulmonary embolism as the primary diagnosis; (b) ability to answer and comprehend the questionnaires; (c) first episode of pulmonary thromboembolism.

Patients were excluded if there was a presence of psychiatric or cognitive disorders, progressive neurological disorders, organ failure, an inability to cooperate, or an inability to provide informed consent.

### 2.3. Grouping

Participants were divided into two groups according to illness severity, which was evaluated using the Pulmonary Embolism Severity Index (PESI). PESI is a practical clinical prediction rule that has been derived and validated in patients admitted to hospitals with PE [15]. PESI employs eleven predictors derived from medical history and physical examination, obviating the necessity for laboratory tests or imaging procedures. This model reliably stratifies patients into five risk groups and is proposed as a potential tool to guide initial treatment intensity. Patients with scores ≥160 (groups IV and V) were included in the high-severity group (HSG), and patients with scores <160 (groups I, II, and III) were included in the low–moderate severity group (LMSG).

### 2.4. Outcome Measures

Patients were evaluated at hospital admission and at 1-month and 3-month follow-up. After hospital discharge, patients continued their daily lives and followed their standard medical treatments.

Anthropometric and sociodemographic data, information about the radiological extension of PE, and the presence of deep venous thrombosis were extracted from patients’ medical records and gathered through patient interviews at hospital admission. In addition, comorbidities were assessed using the Charlson index [16].

Main outcomes were functionality, self-perception of occupational performance, pain, fatigue, and health-related quality of life.

#### 2.4.1. Functionality

Functionality was evaluated by the World Health Organization Disability Assessment Schedule 2.0 (WHODAS 2.0) [17]. Individuals are prompted to indicate the difficulty they have encountered in performing specific tasks in the past 30 days. The WHODAS comprises 36 items across 6 domains (cognition, mobility, self-care, relations, life activities, including housework and work/school activities, and participation). Each domain score ranges from 1 “no difficulty” to 5 “extreme difficulty/can’t do”. Higher scores indicate greater levels of functional impairment. The internal consistency reliability of the WHODAS, which is 2.0, with a Cronbach’s alpha coefficient of 0.7, was established across the four domains related to chronic diseases.

#### 2.4.2. Self-Perception of Occupational Performance

The self-perception of occupational performance was assessed by the Canadian Occupational Performance Measure (COPM) [18]. During a semi-structured interview, patients were prompted to explore areas of activity where they may encounter challenges that they might have to, wish to, or are anticipated to engage in regularly. Participants assess their perceived performance and satisfaction with each chosen activity on a 10-point scale. Higher ratings signify greater significance, improved performance, and heightened satisfaction.

#### 2.4.3. Pain and Fatigue

Pain and fatigue were assessed using a Numeric Rating Scale (NRS). The NRS is an 11-point scale where 0 is no pain/fatigue and 10 is the worst imaginable pain/fatigue [19,20].

#### 2.4.4. Health-Related Quality of Life

The European Quality of Life-5 Dimensions (EQ-5D) was used to assess the health-related quality of life. The EQ-5D has five dimensions: mobility, self-care, daily activities (work, study…), pain/discomfort, and anxiety/depression. These questions are scored between 1 and 5, where 1 represents “no problems” and 5 refers to “extreme problems”. In addition, the scale includes a thermometer-like visual analog scale from 0 to 100, where 0 represents “the worst imaginable state of health” and 100 represents “the best imaginable state of health” [21,22].

### 2.5. Statistical Analysis

A priori power analysis with G*Power 3.1.9.2 software was performed based on a pilot study (unpublished) of fifteen subjects (effect size of 0.50), obtaining a statistical power of 95% and a sample size of 144. However, 159 participants were recruited to allow for a dropout rate of 10% [23].

Statistical Package SPSS version 23.0 (International Business Machines, Armonk, NY, USA) was used to analyze the data obtained. Before statistical analysis, the Kolmogorov–Smirnov test was performed to assess the normality of the variables. Descriptive statistics (i.e., mean ± standard deviation) were carried out to describe sample baseline characteristics. A between-group comparison was performed using Student’s t-test after subjects were grouped by PESI scores. Statistical significance was accepted at a *p*-value of 0.05.

## 3. Results

**Figure 1:**
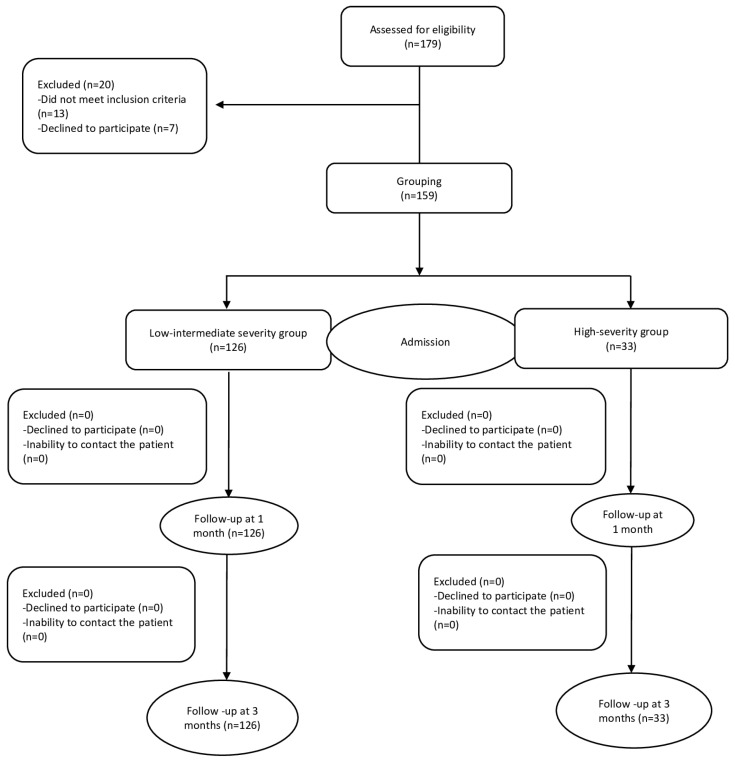
Flow diagram of participants.

A flowchart of participant selection and follow-up is shown in Figure 1.

Of the 179 patients eligible for inclusion in this study, 20 were excluded; 13 did not meet the inclusion criteria and 7 declined to participate in this study. A total of 159 patients were grouped based on clinical severity. Finally, 126 patients were included in the low–moderate severity group and 33 patients were included in the high-severity group (see Figure 1).

Characteristics of the sample at hospital admission are presented in Table 1.

In Table 1, significant differences were observed in terms of age (*p* < 0.05), which was higher in patients included in the high-risk group, and in terms of comorbidities assessed by the Charlson index (*p* < 0.05), with higher rates recorded in patients with PESI > 106. Nevertheless, no significant differences were found between the groups in other baseline characteristics.

Main outcomes at hospital admission are shown in Table 2.

As seen in Table 2, the patients in the high-severity group presented worse scores in most WHODAS domains compared to the patients in the low–moderate severity group: this includes self-care (*p* = 0.013), housework (*p* = 0.023), work and school activities (*p* = 0.008), participation (*p* = 0.025), and total score (*p* < 0.001). In addition, patients with high disease severity reported significantly more pain compared to those with lower disease severity (*p* = 0.041).

Concerning the self-perceived health status of the patients at hospitalization, the group with high disease severity presented significantly worse scores in the mobility, self-care, pain, and anxiety/depression domains and in the VAS (*p* < 0.05)

In the rest of the functional outcome variables, the results were not statistically significantly different between the two groups.

The evolution of outcomes at one month and three months is presented in Table 3.

Statistically significant differences in WHODAS scores (all the subscales except the relations subscale and the total score) (*p* < 0.05) were found between the groups at 1- and 3-month follow-up, in favor of the low–moderate severity group. In addition, patients in the low–moderate severity group showed significant better scores in Self-perception of occupational performance compared to the high-severity group (*p* < 0.05).

At 1- and 3-month follow-up, participants in the high-severity group showed significant impairments in pain, fatigue, and self-perceived health status compared to the other group (*p* < 0.05).

## 4. Discussion

This study aimed to evaluate the relationship between clinical severity and functionality, occupational performance, and health-related quality of life in patients hospitalized with a pulmonary embolism. The results show that there were significant differences in functionality, occupational performance, and health-related quality of life between patients whose thromboembolism was of high severity and patients with thromboembolism of low-to-moderate severity.

The average age of the participants included in our study closely aligns with that of other previously published studies [24,25,26]. This consistency in age demographics suggests comparability across research findings, providing a solid basis for further analysis and interpretation within the broader context of the field. Previous scientific studies have consistently identified a correlation between the severity of pulmonary embolism and the age of patients. Cefalo et al. (2015) [27] carried out a comparison of patients diagnosed with pulmonary embolism who were ≥65 and <65 years old. They concluded that elderly patients present with more severe pulmonary embolism. Our results are in line with this, as patients in the low-to-moderate-severity group had a mean age of 60, whereas those in the high-severity group had a mean age of 75.62.

The majority of the participants were female, which was more pronounced in the high-severity group. These results are in line with a study by Zhang et al. (2023) [28]. In their systematic review and meta-analysis, they concluded that women have high-risk PE more frequently than men.

The participants included in this study were overweight in both groups, with a mean BMI of 28. The correlation between an BMI greater than 24.9 and the incidence of pulmonary embolisms has been extensively explored in the scientific literature [29,30]. This association is often attributed to obesity-related factors such as inflammation, impaired fibrinolysis, and endothelial dysfunction [31].

Regarding hospital stay, our results show significant differences between both groups; patients with high-severity PE were hospitalized for significantly more days compared to patients with low–moderate severity. Hospital stay duration has been correlated to physical deconditioning, psychological distress, and decreased independence in activities of daily living [32].

The decline in quality of life and functional outcomes among patients with PE has been studied previously. Klok et al. (2010) [33] compared functionality and quality of life in patients with PE and sex- and age-matched healthy controls. They found that patients with PE saw a significant deterioration in these variables in the long term. In addition, Farmakis et al. (2023) found that three months after PE, 37% of patients reported dyspnea and 22% had abnormal exercise capacity [34]. Nevertheless, they did not evaluate health-related quality of life and did not compare the results to other groups. Evaluating quality of life is very important in these patients. Kellet et al. (2019) concluded that a reduced quality of life in survivors of an acute PE episode was associated with an increased risk of long-term mortality after a median observation period of 3.6 years [35].

In addition, Farmakis et al. 2023 [36] analyzed the association of exercise capacity with clinical, echocardiographic, and laboratory abnormalities and quality of life after PE. They concluded that 3 and 12 months after acute PE, there was an impairment in exercise capacity that was related to clinical and hemodynamic impairment and to long-term quality of life reduction.

Assessing the quality of life and functionality of patients with PE is crucial for understanding the holistic impact of the condition and guiding comprehensive care strategies.

This study has some limitations. One limitation is that the duration of the follow-up period was limited. Another limitation of our study lies in the utilization of questionnaires rather than objective measures. While questionnaires offer valuable insights into participants’ subjective experiences, attitudes, and perceptions, they may introduce biases and limitations inherent in self-reporting. However, it is essential to acknowledge that subjective measures are also crucial for capturing individuals’ perspectives and understanding complex phenomena from their point of view. In addition, in future studies, incorporating a combination of subjective and objective measures could provide a more comprehensive understanding of the topic, balancing both quantitative and qualitative aspects of research. In addition, future studies should include patients’ history and socioeconomical variation, as well as clinical data at mid-term.

## 5. Conclusions

We concluded that there is an association between clinical severity and mid-term functionality, self-perception of occupational performance, pain, fatigue, and health-related quality of life in patients with PE.

These findings underscore the importance of the early detection and appropriate management of pulmonary thromboembolism to minimize its detrimental impact on patients’ overall health and functioning.

## Figures and Tables

**Table 1:** Characteristics of the sample at hospital admission.

Variable		Low–Moderate Severity Group (*n* = 126)	High-Severity Group (*n* = 33)	*p*-Value
Hospitalization stay (days)		6.19 ± 8.46	10.94 ± 12.48	0.706
Sex (% men)		52.5	39.1	0.562
Age (years)		60.11 ± 15.30	75.62 ± 12.17	0.045 *
BMI (Kg/m^2^)		28.65 ± 4.92	28.32 ± 4.09	0.223
Charlson index		3.17 ± 2.28	6.21 ± 2.40	0.043 *
Presence of TVP (%)		59	40	0.711
Extension of PE (%)	unilateral central	5.1	8	0.394
	bilateral central	55.9	48	
	unilateral peripheral	11.9	12	
	Bilateral peripheral	27.11	24	

BMI: Body Mass Index; TVP: deep vein thrombosis; PE: pulmonary embolism. Variables are expressed as mean ± standard deviation or percentage (%). * *p* < 0.05.

**Table 2:** Main outcomes at hospital admission.

Variable		Low–Moderate Severity Group (*n* = 126)	High-Severity Group (*n* = 33)	*p*-Value
WHODAS	Cognition	8.14 ± 4.26	8.82 ± 3.72	0.872
	Mobility	9.41 ± 6.20	10.91 ± 5.93	0.652
	Self-care	5.62 ± 3.91	9.10 ± 4.85	0.013 *
	Relations	6.76 ± 3.30	6.91 ± 3.04	0.829
	Housework	7.29 ± 5.28	10.54 ± 6.86	0.023 *
	Work and school activities	9.61 ± 6.89	12.87 ± 8.32	0.008 *
	Participation	14.12 ± 7.41	17.27 ± 7.85	0.025 *
	Total	58.43 ± 28.82	72.27 ± 32.37	<0.001 **
COMP test	Satisfaction	7.89 ± 4.41	6.23 ± 3.89	0.763
	Performance	3.50 ± 3.53	2.06 ± 2.39	0.845
NRS pain		3.16 ± 3.22	2.70 ± 3.12	0.041 *
NRS fatigue		3.12 ± 2.25	3.20 ± 3.27	0.429
EQ-5D	Mobility	2.00 ± 1.22	2.63 ± 1.24	0.003 *
	Self-care	2.02 ± 1.27	2.36 ± 1.64	0.028 *
	Daily activities	2.14 ± 1.31	2.64 ± 1.39	0.053
	Pain/discomfort	1.69 ± 0.80	1.73 ± 1.23	0.014 *
	Anxiety/depression	1.48 ± 0.88	1.82 ± 0.95	0.009 *
	EQ VAS	58.72 ± 23.38	53.50 ± 20.85	0.034 *

PE: pulmonary embolism; WHODAS: World Health Organization Disability Assessment Schedule; COMP: Canadian Occupational Performance Measure; NRS: Numeric Rating Scale; EuroQol: European Quality of Life-5 Dimensions; EQ VAS: Visual Analogue Scale. Variables are expressed as mean ± standard deviation or percentage (%). * *p* < 0.05. ** *p* < 0.001.

**Table 3:** Main outcomes at 1-month and 3-month follow-up.

Variable			Low–Moderate Severity Group (*n* = 126)	High-Severity Group (*n* = 33)	*p*-Value
WHODAS	Cognition	1 month	7.44 ± 2.47	12.00 ± 9.00	<0.001 **
		3 months	10.13 ± 6.48	8.22 ± 2.34	0.002 *
	Mobility	1 month	8.37 ± 5.96	14.67 ± 6.61	<0.001 **
		3 months	9.90 ± 4.96	7.67 ± 2.84	0.035 *
	Self-care	1 month	4.59 ± 2.31	9.33 ± 6.56	<0.001 **
		3 months	5.42 ± 3.46	4.33 ± 0.68	0.012 *
	Relations	1 month	5.85 ± 1.73	6.33 ± 2.00	0.046 *
		3 months	6.61 ± 3.55	6.00 ± 1.44	0.543
	Housework	1 month	6.63 ± 5.01	13.33 ± 7.21	<0.001 **
		3 months	7.93 ± 4.91	6.56 ± 4.02	0.047 *
	Work and school activities	1 month	8.73 ± 7.69	16.00 ± 12.03	<0.001 **
		3 months	7.68 ± 5.40	5.40 ± 2.90	0.039 *
	Participation	1 month	13.00 ± 6.36	24.33 ± 12.32	<0.001 *
		3 months	16.20 ± 7.32	10.44 ± 4.97	<0.001 *
	Total	1 month	49.00 ± 19.53	96.00 ± 49.39	<0.001 *
		3 months	59.34 ± 25.29	45.22 ± 10.58	<0.001 *
COMP test	Satisfaction	1 month	10.73 ± 6.49	9.07 ± 0.36	0.026 *
		3 months	7.83 ± 3.25	4.60 ± 0.00	<0.001 **
	Performance	1 month	7.00 ± 3.18	6.62 ± 0.41	0.035 *
		3 months	7.83 ± 1.64	4.60 ± 0.00	<0.001 **
NRS pain		1 month	2.43 ± 3.23	4.33 ± 3.50	<0.001 **
		3 months	2.62 ± 2.80	2.22 ± 2.79	0.045 *
NRS fatigue		1 month	3.04 ± 3.49	9.00 ± 0.87	<0.001 **
		3 months	3.88 ± 3.60	2.44 ± 3.30	0.038 *
EuroQol (5-D)	Mobility	1 month	1.31 ± 0.67	2.00 ± 0.00	<0.001 **
		3 months	1.31 ± 0.47	1.40 ± 0.50	0.123
	Self-care	1 month	1.19 ± 0.48	1.50 ± 0.55	0.055
		3 months	1.23 ± 0.49	1.20 ± 0.41	0.525
	Daily activities	1 month	1.50 ± 0.85	3.00 ± 0.00	<0.001 **
		3 months	1.31 ± 0.47	1.40 ± 0.50	0.046 *
	Pain/discomfort	1 month	1.50 ± 0.68	1.50 ± 0.58	0.852
		3 months	1.66 ± 0.65	1.60 ± 0.67	0.329
	Anxiety/depression	1 month	1.65 ± 0.88	1.50 ± 0.55	0.425
		3 months	1.34 ± 0.59	1.30 ± 0.47	0.685
	EQ VAS	1 month	78.00 ± 70.00	45.00 ± 5.48	<0.001 **
		3 month	66.58 ± 21.04	78.33 ± 18.29	<0.001 **

PE: pulmonary embolism; WHODAS: World Health Organization Disability Assessment Schedule; COMP: Canadian Occupational Performance Measure; NRS: Numeric Rating Scale; EuroQol: European Quality of Life-5 Dimensions; EQ VAS: Visual Analogue Scale. Variables are expressed as mean ± standard deviation or percentage (%). * *p* < 0.05. ** *p* < 0.001.

## Data Availability

The datasets used in this study are available from the corresponding author on reasonable request.

## References

[1] Goldhaber S.Z., Bounameaux H. (2012). Pulmonary embolism and deep vein thrombosis. Lancet.

[2] Stein P.D., Beemath A., Olson R.E. (2005). Trends in the incidence of pulmonary embolism and deep venous thrombosis in hospitalized patients. Am. J. Cardiol..

[3] Martin K.A., Molsberry R., Cuttica M.J., Desai K.R., Schimmel D.R., Khan S.S. (2020). Time Trends in Pulmonary Embolism Mortality Rates in the United States, 1999 to 2018. J. Am. Heart. Assoc..

[4] Konstantinides S.V., Barco S., Lankeit M., Meyer G. (2016). Management of Pulmonary Embolism: An Update. J. Am. Coll. Cardiol..

[5] Torbicki A., Perrier A., Konstantinides S., Agnelli G., Galiè N., Pruszczyk P., Bengel F., Brady A.J., Ferreira D., Janssens U. (2008). ESC Committee for Practice Guidelines (CPG). Guidelines on the diagnosis and management of acute pulmonary embolism: The Task Force for the Diagnosis and Management of Acute Pulmonary Embolism of the European Society of Cardiology (ESC). Eur. Heart J..

[6] Chan C.M., Woods C., Shorr A.F. (2010). The validation and reproducibility of the pulmonary embolism severity index. J. Thromb. Haemost..

[7] Zhou X.Y., Ben S.Q., Chen H.L., Ni S.S. (2012). The prognostic value of pulmonary embolism severity index in acute pulmonary embolism: A meta-analysis. Respir. Res..

[8] Nordenholz K., Ryan J., Atwood B., Heard K. (2011). Pulmonary embolism risk stratification: Pulse oximetry and pulmonary embolism severity index. J. Emerg. Med..

[9] Sandal A., Korkmaz E.T., Aksu F., Koksal D., Selcuk Z.T., Demir A.U., Emri S., Coplu L. (2021). Performance of pulmonary embolism severity index in predicting long-term mortality after acute pulmonary embolism. Anatol. J. Cardiol..

[10] Hariharan P., Takayesu J.K., Kabrhel C. (2011). Association between the Pulmonary Embolism Severity Index (PESI) and short-term clinical deterioration. Thromb. Haemost..

[11] Klok F.A., van der Hulle T., den Exter P.L., Lankeit M., Huisman M.V., Konstantinides S. (2014). The post-PE syndrome: A new concept for chronic complications of pulmonary embolism. Blood Rev..

[12] Kahn S.R. (2016). The post-thrombotic syndrome. Hematol. Am. Soc. Hematol. Educ. Program..

[13] World Medical Association (2013). World Medical Association Declaration of Helsinki: Ethical principles for medical research involving human subjects. JAMA.

[14] Cuschieri S. (2019). The STROBE guidelines. Saudi J. Anaesth..

[15] Aujesky D., Obrosky D.S., Stone R.A., Auble T.E., Perrier A., Cornuz J., Roy P.-M., Fine M.J. (2005). Derivation and validation of a prognostic model for pulmonary embolism. Am. J. Respir. Crit. Care Med..

[16] Charlson M.E., Pompei P., Ales K.L., MacKenzie C.R. (1987). A new method of classifying prognostic comorbidity in longitudinal studies: Development and validation. J. Chronic Dis..

[17] Üstün T.B., Chatterji S., Kostanjsek N., Rehm J., Kennedy C., Epping-Jordan J., Saxena S., Von Korff M., Pull C. (2010). WHO/NIH Joint Project. Developing the World Health Organization Disability Assessment Schedule 2.0. Bull. World Health Organ..

[18] Law M., Baptiste S., McColl M., Opzoomer A., Polatajko H., Pollock N. (1990). The Canadian occupational performance measure: An outcome measure for occupational therapy. Can. J. Occup. Ther..

[19] Fraenkel L., Falzer P., Fried T., Kohler M., Peters E., Kerns R., Leventhal H. (2012). Measuring pain impact versus pain severity using a numeric rating scale. J. Gen. Intern. Med..

[20] Micklewright D., St Clair Gibson A., Gladwell V., Al Salman A. (2017). Development and Validity of the Rating-of-Fatigue Scale. Sports Med..

[21] Badia X., Roset M., Montserrat S., Herdman M., Segura A. (1999). La versión española del EuroQol: Descripción y aplicaciones [The Spanish version of EuroQol: A description and its applications. European Quality of Life scale. Med Clin..

[22] Feng Y.S., Kohlmann T., Janssen M.F., Buchholz I. (2021). Psychometric properties of the EQ-5D-5L: A systematic review of the literature. Qual. Life Res..

[23] Faul F., Erdfelder E., Lang A.G., Buchner A. (2007). G*Power 3: A flexible statistical power analysis program for the social, behavioral, and biomedical sciences. Behav. Res. Methods.

[24] Jiménez D., Resano S., Otero R., Jurkojc C., Portillo A.K., Ruiz-Artacho P., Corres J., Vicente A., Exter P.L.D., Huisman M.V. (2015). IRYCIS Pulmonary Embolism Study Group. Computerised clinical decision support for suspected PE. Thorax.

[25] Friera-Reyes A., Caballero P., Ruiz-Giménez N., Artieda P., Domínguez L., Pérez-Amor E., Suárez C., Grupo de Estudio de Enfermedad Tromboembólica Venosa (2005). Utilidad del dímero-D por ELISA rápido en el diagnóstico de la embolia pulmonar en un servicio de urgencias [Usefulness of fast ELISA determination of D-dimer levels for diagnosing pulmonary embolism in an emergency room]. Arch. Bronconeumol..

[26] Acar H., Yılmaz S., Yaka E., Doğan N.Ö., Özbek A.E., Pekdemir M. (2017). Evaluation of the Diagnostic Role of Bedside Lung Ultrasonography in Patients with Suspected Pulmonary Embolism in the Emergency Department. Balkan Med. J..

[27] Cefalo P., Weinberg I., Hawkins B.M., Hariharan P., Okechukwu I., Parry B.A., Chang Y., Rosovsky R., Liu S.W., Jaff M.R. (2015). A comparison of patients diagnosed with pulmonary embolism who are ≥65 years with patients <65 years. Am. J. Cardiol..

[28] Zhang Y., Qiu Y., Luo J., Zhang J., Yan Q. (2023). Sex-Based Differences in the Presentation and Outcomes of Acute Pulmonary Embolism: A Systematic Review and Meta-Analysis. Tex. Heart Inst. J..

[29] Grimnes G., Isaksen T., Tichelaar Y.I.G.V., Brox J., Brækkan S.K., Hansen J.B. (2018). C-reactive protein and risk of venous thromboembolism: Results from a population-based case-crossover study. Haematologica.

[30] Ageno W., Becattini C., Brighton T., Selby R., Kamphuisen P.W. (2008). Cardiovascular risk factors and venous thromboembolism: A meta-analysis. Circulation.

[31] Gérard D., Czernichow S., Salomon L., Carette C. (2019). Impact of obesity on the prognostic value of the ventilation/perfusion scan in acute pulmonary embolism. Respirology.

[32] Patel N., Smith C.E., Pinilla M., Schmidt R.J. (2019). The association between length of hospitalization and functional improvement among elderly hip fracture patients. Int. J. Orthop. Trauma.

[33] Klok F.A., van Kralingen K.W., van Dijk A.P., Heyning F.H., Vliegen H.W., Kaptein A.A., Huisman M.V. (2010). Quality of life in long-term survivors of acute pulmonary embolism. Chest.

[34] Farmakis I.T., Valerio L., Barco S., Christodoulou K.C., Ewert R., Giannakoulas G., Held M., Hobohm L., Keller K., Wilkens H. (2024). Functional capacity and dyspnea during follow-up after acute pulmonary embolism. J. Thromb. Haemost..

[35] Keller K., Tesche C., Gerhold-Ay A., Nickels S., Klok F.A., Rappold L., Hasenfuß G., Dellas C., Konstantinides S.V., Lankeit M. (2019). Quality of life and functional limitations after pulmonary embolism and its prognostic relevance. J. Thromb. Haemost..

[36] Farmakis I.T., Valerio L., Barco S., Alsheimer E., Ewert R., Giannakoulas G., Hobohm L., Keller K., Mavromanoli A.C., Rosenkranz S. (2023). Cardiopulmonary exercise testing during follow-up after acute pulmonary embolism. Eur. Respir. J..

